# Plant hexokinase phylogenetic analysis highlights a possible regulation by the posttranslational modifier SUMO

**DOI:** 10.17912/micropub.biology.000260

**Published:** 2020-06-29

**Authors:** Pedro Humberto Castro, Sara Freitas, Herlander Azevedo

**Affiliations:** 1 CIBIO, InBIO - Research Network in Biodiversity and Evolutionary Biology, Universidade do Porto, Campus Agrário de Vairão, 4485-661 Vairão, Portugal; 2 Departamento de Biologia, Faculdade de Ciências, Universidade do Porto, Rua Campo Alegre, 4169-007 Porto, Portugal

**Figure 1:**
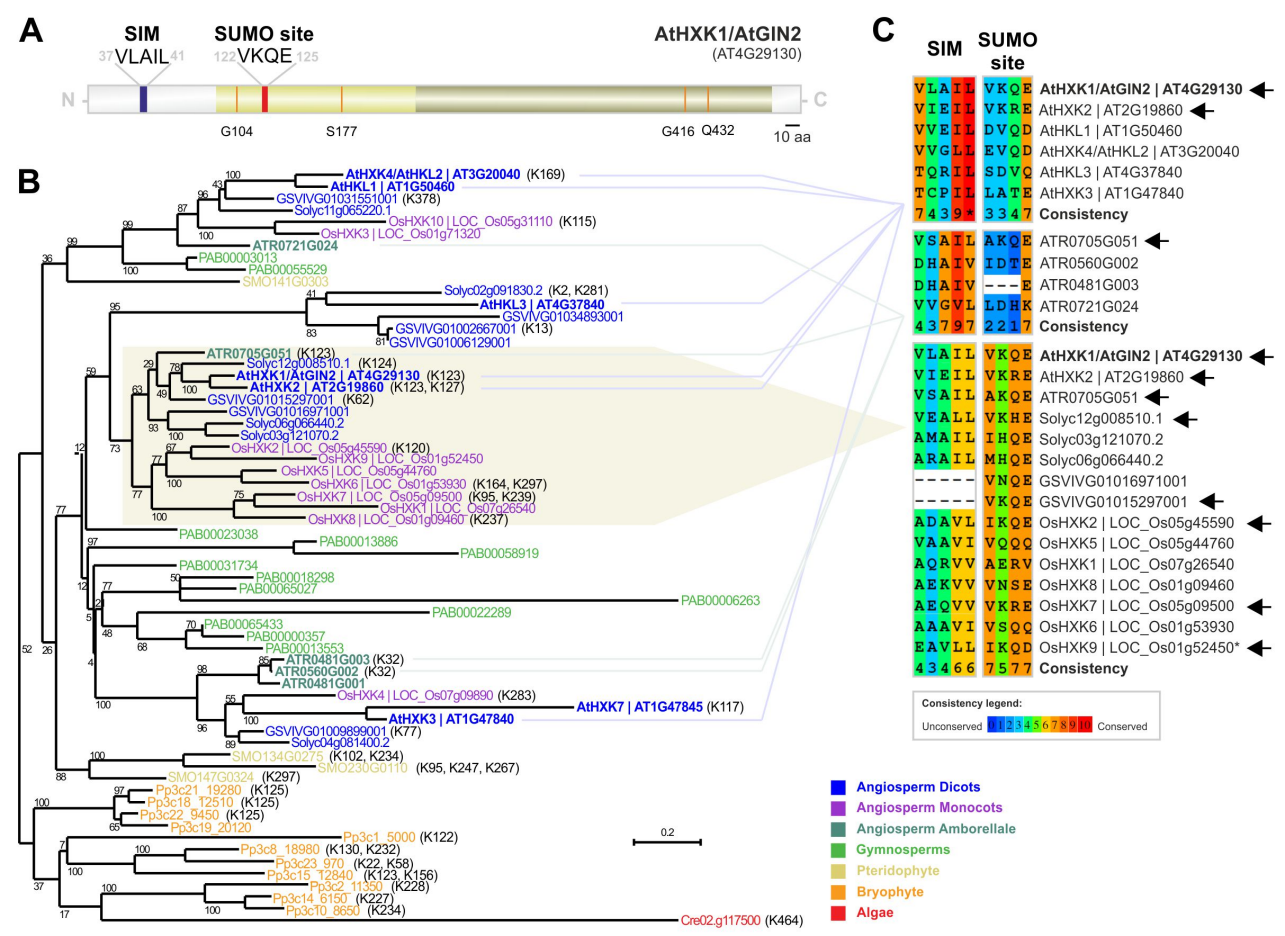
Hexokinase SUMO site and SUMO-interacting motif (SIM) prediction and evolutionary conservation in plants. (**A**) Predicted sumoylation and SIM sites are indicated in red and blue, respectively. Light yellow and brown correspond to hexokinase small and large subdomains, respectively. The orange colour indicates the residues that were previously determined to have a role in AtHXK1 functioning (Moore *et al.*, 2003). Scale bar indicates 10 amino acids (aa). (**B**) The plant Hexokinase phylogenetic tree was generated using RaxML with 1000 bootstrap values (numbers represent the bootstrap percentage). Lysines with high or medium probability of sumoylation, inferred bioinformatically using GPS-SUMO 1.0, are indicated between brackets. The prefix for each sequence corresponds to prefixes for GeneIDs as implemented in PLAZA (Van Bel *et al.*, 2018), and represent the following species: At (*Arabidopsis thaliana,* ARATH), Solyc (*Solanum lycopersicum*, SOLYC), GSVIV (*Vitis vinifera*, VITVI), LOC*_*Os and Os (*Oryza sativa* ssp. *Japonica*, ORYSJ), ATR (*Amborella trichopoda*, AMBTC), PAB (*Picea abies*, PICAB), SMO (*Selaginella moellendorffii*, SELML), Pp (*Physcomitrella patens*, PHYPA), and Cre (*Chlamydomonas reinhardtii*, CHLRE). (**C**) Multiple sequence alignment of SUMO and SIM sites in *Arabidopsis thaliana*, *Amborella trichopoda*, and group 6/7 HXKs. Amino acid consistency is classified from 0 (unconserved) to 10 (conserved). Arrows indicate motifs with predicted SUMO-targeted lysine. Asterisk indicates a SUMO site predicted by GSP-SUMO to be low probability rather than high/medium probability. The first set of alignments corresponds to *Arabidopsis thaliana* HXKs (excluding the truncated AtHXK7). The second set of alignments was produced with *Amborella trichopoda* sequences (excluding the truncated ATR0481G001). The last panel of alignment was produced using members from HXK group 6 and 7, as classified by Karve *et al.* (2010).

## Description

Hexokinases (HXKs) are central enzymes in the carbohydrate metabolism, by controlling the entry point of glucose into glycolysis, while also displaying regulatory roles. In plants, *Arabidopsis thaliana* HXK1 (AtHXK1, also known as AtGIN2) is involved in hormonal signalling, which likely explains its involvement in plant growth and development (Moore *et al.*, 2003). One interesting feature of AtHXK1 is that, in the presence of exogenous glucose, it localizes in the nucleus to form a complex that inhibits photosynthesis-associated transcripts (Cho *et al.*, 2006). This feature is central to cope with excess sugar by diminishing sugar biosynthesis. With such a central role, it is likely that AtHXK1 has several layers of regulation, and it is therefore logical that AtHXK1 may be targeted by different posttranslational modifications (PTMs). In support, PTMs such as phosphorylation and ubiquitination have been proposed to control HXK activity in human cells (Zhang *et al.*, 2017; Lee *et al.*, 2019). At the plant level however, the knowledge concerning HXK regulation is very scarce.

In a previous work, it was reported that Arabidopsis HXK1 interacts with Arabidopsis SUMO E2 conjugase (SCE1), based on a SCE1-bait yeast-two-hybrid screening assay (Elrouby and Coupland, 2010). This places HXK1 as a potential target for the peptide small ubiquitin-like modifier (SUMO). Conversely, we genetically demonstrated, using null mutants for HXK1 and the central SUMO pathway component SIZ1, that both genes fail to act epistatically in the control of plant growth (Castro *et al*. 2020).SUMO covalent attachment to a protein, known as sumoylation, normally occurs on a lysine within a consensus motif ΨKXE (Ψ, large hydrophobic residue; K, lysine; X, any amino acid; E, glutamic acid) of a target protein. Sumoylation follows a multi-step enzymatic cascade that includes deSUMOylating proteases (DSP)-dependent maturation of the preSUMO peptide, E1 activation, and E2 conjugation, normally facilitated by an E3 ligase. In addition, SUMO may also interact non-covalently with a protein, more specifically with a target’s SUMO-interacting motif (SIM, a hydrophobic amino acids stretch flanked by acidic residues) (Gareau and Lima, 2010). SUMO may control a target’s activity on multiple levels, including regulation of subcellular localization, modulation of protein interactions and complex assembly, changes in enzymatic activity, and potentiation/antagonization of other PTMs (Augustine and Vierstra, 2018).

Here, we explore Arabidopsis HXK1 potential sumoylation sites, extended to other plant species to inspect the levels of evolutionary conservation. First, we submitted the Arabidopsis HXK1 protein sequence to the sumoylation site and SUMO-interaction site predictor software GPS-SUMO 1.0 (Zhao *et al.*, 2014). This software predicted a high confidence sumoylation site in K123 and a SIM site (VLAIL motif) at position 37-41 (Fig. 1A). This prediction supports the hypothesis that sumoylation and/or SUMO interaction regulates HXK activity. Interestingly, the SUMO site is inside the predicted Hexokinase small subdomain, whereas the SIM is outside and closer to the N-terminus. To investigate if these motifs were conserved, indicating a conserved role for SUMO-HXK among plants, we performed a phylogenetic study in plant HXKs (Fig. 1B). HXK1 belongs to a multigene family that branched and expanded across plant evolution (Karve *et al.*, 2010). While only one member exists in the algae *Chlamydomonas reinhardtii*, several HXKs are present in higher plants, ranging from four in the pteridophyte *Selaginella moellendorffii*, up to 13 members in the gymnosperm *Picea abies* (Fig. 1B). In Arabidopsis we observed the presence of seven members, one more than previous studies suggested (Karve *et al.*, 2010). This additional protein, with AGI code AT1G47845, was probably overlooked in previous phylogenetic studies because of its truncated topology. None withstanding, the PFAM software predicted the existence of an HXK domain. In line with the previous AtHXKs naming order, we decided to designate AT1G47845 as AtHXK7.

Next we performed an alignment of Hexokinase members to inspect the level of conservation of AtHXK1’s K123 and SIM in all Arabidopsis paralogs (Fig. 1C, first panel). AtHXK7 was excluded from the analysis for being truncated in this region. Among Arabidopsis HXKs, only AtHXK1 (K123) and AtHKX2 (K123) revealed to have this lysine. AtHXK1 and AtHXK2 are members of the group 6 (Karve *et al.*, 2010). Interestingly, the basal angiosperm *Amborella trichopoda* presented a member, ATR0705G051, clustered with AtHXK1/AtHXK2, that also contains a lysine (K123) with high/medium probability of sumoylation (Fig. 1C, second panel). In other *Amborella trichopoda* HXKs the lysine amenable to sumoylation exists but is in a different region of the protein (Fig. 1B,C). The fact that *Amborella trichopoda* protein ATR0705G051 clustered with dicots means that the Karve *et al.* (2010) classification into groups 6 and 7 (the latter only displaying monocots), should in fact be reconsidered as a single phylogenetic group. To evaluate if this lysine was conserved in group 6/7 we performed an alignment with all its members (Fig. 1C, third panel). Albeit not all HXKs within the group 6/7 contain the conserved motif, it is important to highlight that at least one HXK with the expected lysine exists in each species (Fig. 1C). In fact, the same lysine seems to be conserved across plant HXKs, being detected also in bryophytes and pteridophytes (Fig. 1B). The exception is the gymnosperm *Picea abies*, but this may derive from miss-annotation given the complexity of this genome. Results suggest that each species tends to retain at least one homologous K123 amenable to sumoylation during evolution.

The sumoylation of AtHXK1 was previously tested in bacteria, using an *E. coli* expressing a heterologous sumoylation system. AtHXK1 was positively sumoylated by both AtSUM1 and AtSUM3 isoforms (Elrouby and Coupland, 2010). However, the biological relevance of AtHXK1 sumoylation is yet to be determined. Nevertheless, we can discuss plausible scenarios. For instance, SUMO is involved in nuclear-cytoplasm trafficking and nuclear complex assembly (Palancade and Doye, 2008; Jentsch and Psakhye, 2013). The N-terminus of the human glucokinase (GK) is multi-sumoylated, which enhances glucose uptake, glycolysis and glycogen synthesis upon glucose treatment (Aukrust *et al.*, 2013). Aukrust *et al.* (2013) proposed that SUMO contributes to GK activity by controlling the translocation between the cytoplasm and nucleus via the nucleoporin SUMO E3 ligase RANBP2. AtHXK1 is normally located in the mitochondria (Balasubramanian *et al.*, 2007) but also, due to HXK1’s sugar signalling role, it can be relocated to the nucleus as part of a transcription repressor complex (Cho *et al.*, 2006). In contrast, SUMO is majorly nuclear-located, but can also be detected in the cytoplasm (Lois *et al.*, 2003; Saracco *et al.*, 2007). It would be interesting to determine if SUMO can control AtHXK1 subcellular localization and if that has an impact on AtHXK1 activity. On the other hand, using GFP fusion constructs with OsHXK5 and OsHXK6, revealed that both retain a dual subcellular localization to mitochondria and the nucleus (Cho *et al.*, 2009) and none of these contain the homologous K123. Another likely scenario is that SUMO may also interfere with protein-protein interactions or the assembly of protein complexes. A previous screening for HXK1-interacting partners identified the SNF1-related protein kinase (SnRK) regulatory subunit gamma 1 (SnRK1γ/KINγ) (Van Dingenen *et al.*, 2019). SnRK1 is also a key regulator of cellular sugar and energy homeostasis of the cell (Crozet *et al.*, 2014), and interestingly many of its subunits are modified and regulated by SUMO (Crozet *et al.*, 2016). Increasing evidence points towards a strong SUMO-carbohydrate crosstalk (Castro *et al.*, 2015; Castro *et al.*, 2018b), and future efforts are necessary to uncover weather SUMO plays a role in HXK activity, and conversely determine the role of HXKs in SUMO-dependent carbohydrate signalling/metabolism regulation.

## Methods

The AtHXK1/AtGIN2 sequence was retrieved from the database Plaza Dicots 4.0 (Van Bel *et al.*, 2018) and submitted to Quick Scan mode of ScanProsite (de Castro *et al.*, 2006) to scan for motifs/domains. To determine HXK sumoylation and SUMO-interaction sites, protein sequences were submitted for analysis in the software GPS-SUMO 1.0 (Zhao *et al.*, 2014). The phylogeny of the plant Hexokinase gene family was carried out using the strategy previously described by Castro *et al.* (2018a). Amino acid sequences were retrieved using a combination of automated gene family assignment and curated homology analysis. More specifically, homologs of nine different species were retrieved from the comparative genomics database Plaza Dicots 4.0 (Van Bel *et al.*, 2018), queried using the AGI code for AtHXK1/AtGIN2 (AT4G29130). Species were selected to include all major plant taxa (Bryophyte, Pteridophyte, Gymnosperms, the basal Angiosperm *Amborella trichopoda*, Angiosperm Monocots and Angiosperm Eudicots including the model *Arabidopsis thaliana*). The ancestral Algae species, *Chlamydomonas reinhardtii*, was used as outgroup. Sequences indicated by the Plaza database as outliers were excluded from the analysis. For all protein sequences, we detected the presence of Pfam protein domains in a batch search using HMMSCAN (https://www.ebi.ac.uk/Tools/hmmer/search/hmmscan) and confirmed them to have an hexokinase domain. Amino acid sequences were aligned using MAFFT (v7.402) on CIPRES (Katoh and Standley, 2013). We tested for the substitution best-fit model using ProtTest v3.3 (Darriba *et al.*, 2011). The phylogenetic tree was estimated using maximum likelihood (RaxML v8.2.12 on CIPRES) with a JTT substitution model and 1000 bootstrap iterations. Computation was run at the CIPRES Science Gateway V3.3 (http://www.phylo.org) (Miller *et al.*, 2011). The tree was visualized using SeaView v4.4.0 (Gouy *et al.*, 2010). Sequence alignments for specific sets of proteins were conducted using PRALINE (Simossis and Heringa, 2005). To determine gene nomenclature/designation, Arabidopsis and rice gene IDs were submitted to _at to AGI Conversion Tool (http://bar.utoronto.ca/ntools/cgi-bin/ntools_agi_converter.cgi) and Rice Genome Database Project (http://rice.plantbiology.msu.edu/), respectively.
